# Hypogammaglobulinemia factitia- Munchausen syndrome masquerading as common variable immune deficiency

**DOI:** 10.1186/1710-1492-9-36

**Published:** 2013-09-17

**Authors:** Rohan Ameratunga, Paul Casey, Susan Parry, Chris Kenedi

**Affiliations:** 1 Department of Clinical Immunology, Auckland Hospital, Park Rd, Grafton, Auckland 1010, New Zealand; 2 Department of Gastroenterology, Middlemore Hospital, Auckland, new Zealand; 3 Departments of Liaison psychiatry and General Medicine, Auckland Hospital, Auckland, New Zealand; 4 Department of Internal Medicine, Duke University Medical Center, Durham, NC, USA; 5 Department of Psychiatry, Duke University Medical Center, Durham, NC, USA

**Keywords:** Factitious disorder, NSAID, CVID, Hypogammaglobulinemia factitia, Munchausen syndrome

## Abstract

**Background:**

We describe the first case of a patient with factitious disorder who closely simulated a primary immune deficiency disorder – Common Variable Immune Deficiency (CVID), by surreptitiously ingesting non-steroidal anti-inflammatory agents.

**Case description:**

He was treated with several expensive and potentially dangerous drugs before the diagnosis was established through collateral information. In retrospect he did not meet the proposed new criteria for CVID. These criteria may prove useful in distinguishing cases of CVID from secondary hypogammaglobulinemia.

**Conclusion:**

It is imperative clinicians recognise patients with factitious disorder at the earliest opportunity to prevent iatrogenic morbidity and mortality.

## Introduction

Patients with factitious disorder have feigned a tremendous variety of physical and mental illnesses and it can be very difficult for clinicians to distinguish between real and fabricated symptoms. Therefore, most specialists in the field of factitious disorders, such as psychosmatic or consult-liaison psychiatrists look for a pattern of behaviour rather than a single triggering finding to make the diagnosis. For specialists who do not knowingly encounter these patients, the correct diagnosis of factitious disorder may never be made because it is not even considered. People with factitious disorder deceive clinicians in order to be treated as patients. Severe cases of factitious disorder are sometimes referred to as Munchausen syndrome [1].

The condition may involve the exaggeration or false reports of symptoms of an actual underlying disease or the complete fabrication of subjective complaints, physical exam and/or laboratory findings. By definition, these patients are not seeking a secondary gain such as money, time off work or recreational drugs. The focus of these patients is to be cared for and for most of these patients it is probably a maladaptive coping mechanism to manage their emotional distress. Simulating the disease can be painful and even dangerous for these patients. Factitious behaviour often results in unnecessary medical or surgical interventions that carry risks for the health or even the life of the patient.

Studies suggest these patients account for 0.2-1.0% of hospital admissions but this may be an underestimate as the disorder is often unsuspected [2]. A study from the NIH [3] found that patients with factitious fevers accounted for 9.3% of patients referred to a tertiary hospital for fever of unknown origin. Patients with factitious disorder have presented for investigation of secondary immunological disorders including HIV/AIDS [4] and abscesses from self-injection of fecal material [5]. Two cases of suspected Munchausen syndrome by proxy were described in a case series of children with intestinal pseudo obstruction and hypogammaglobulinemia [6]. We present the first known case of a patient with factitious disorder who was treated for severe hypogammaglobulinemia and Common Variable Immune Deficiency (CVID).

## Case report

A male in his 30s was seen as an outpatient at a large metropolitan hospital with a seven month history of a 20 kg weight loss, pallor and lethargy. He had developed chronic diarrhea. Further investigations confirmed the presence of severe anemia (Hb 40 g/l, nr >125) and profound hypoalbuminemia (23 g/l, nr >40). On initial presentation he minimized his medical history and noted he had conceived a child with in-vitro fertilization 2 years prior to his symptoms.

**Figure 1:**
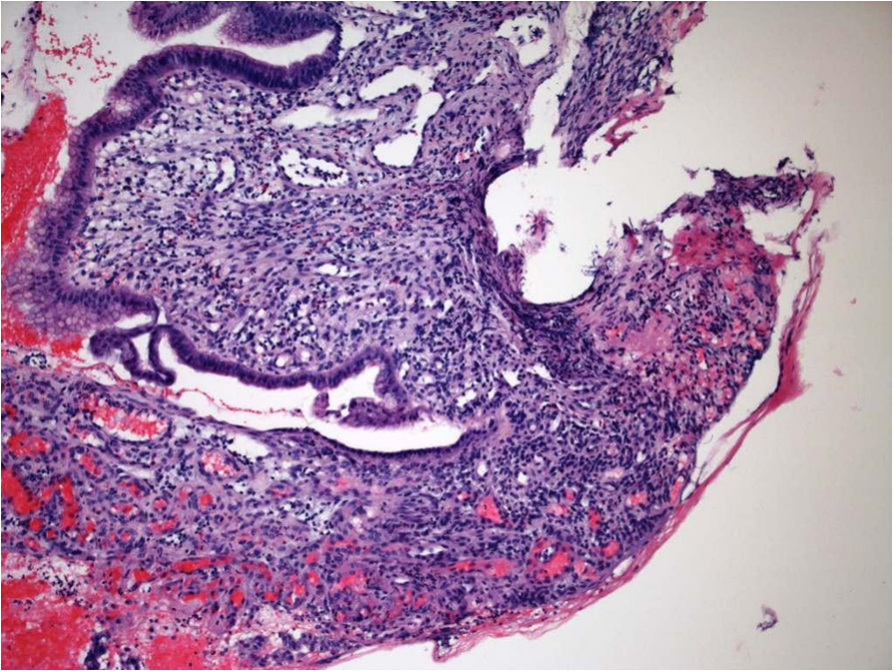
Duodenal biopsy showing ulceration from NSAID abuse.

Gastroscopy showed duodenal atrophy consistent with celiac disease. Duodenal ulceration was also noted (Figure 1). The biopsies showed villous atrophy but absence of a lymphocyte infiltrate. A diagnosis of presumptive celiac disease was made in spite of the absence serological markers. He was placed on a gluten free diet. His anemia was thought to be due to iron malabsorption and he was treated with a blood transfusion and an infusion of parenteral iron. He was taking nonsteroidal anti-inflammatory drugs (NSAIDS) for a knee injury and was advised to discontinue these.

A colonoscopy was normal. A CT scan of the abdomen did not reveal malignancy. He had a markedly elevated fecal calprotectin (>500 μg/g feces, nr <50) indicating ongoing small intestine inflammation. His ESR and CRP were elevated. There was no improvement in his clinical state with ongoing anemia and lethargy despite a gluten free diet and reporting no further NSAID use.

**Figure 2:**
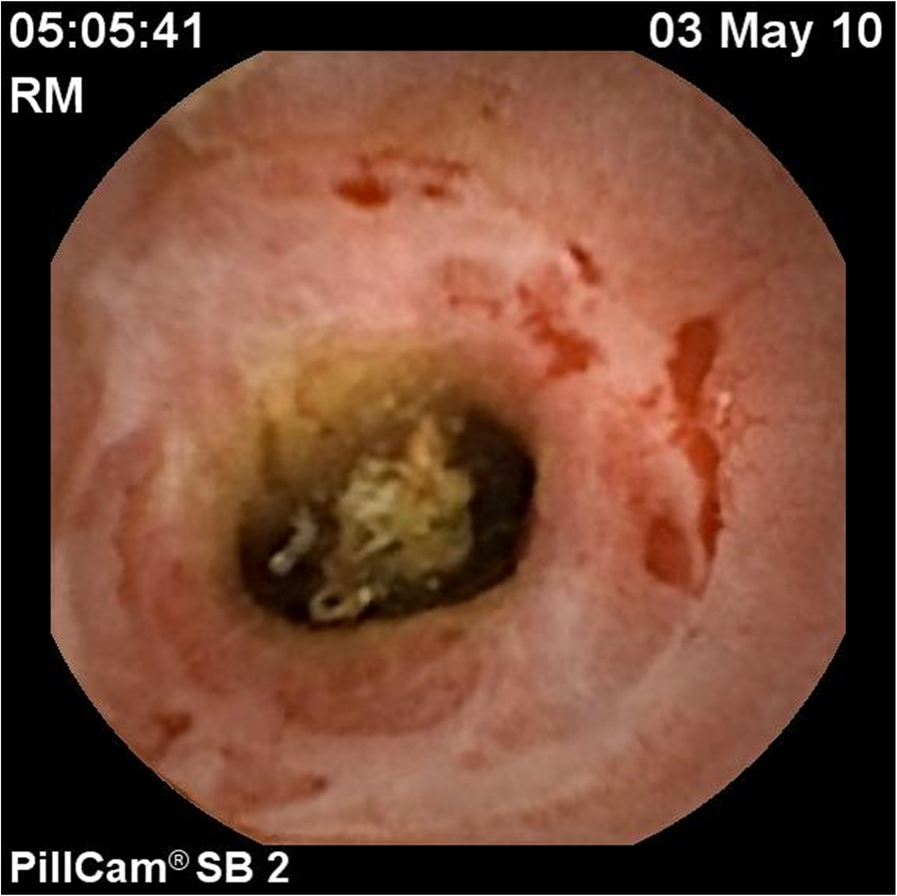
Capsule endoscopy showing extensive enteritis of the jejunum.

Capsule endoscopy was undertaken which showed extensive, severe ulceration of the small intestine (Figure 2). He was thought to have concomitant Crohn’s disease. His ASCA antibodies were negative. In the context of clinical decline despite gluten free diet, the patient’s confirmation of NSAID withdrawal and extensive small bowel ulceration on capsule study, he was treated with high dose oral prednisone, with temporary improvement of his symptoms. Azathioprine was added as a steroid sparing agent.

His immunoglobulins were subsequently measured, revealing profound hypogammaglobulinemia. His IgG was 1.5 g/l (nr> 8) with reduced IgA of 0.4 g/l (nr> 0.8) but IgM just in the normal range at 0.4 g/l (nr> 0.4). He was referred to the clinical immunology service at Auckland Hospital with suspected Common Variable Immune Deficiency (CVID) with predominant gut involvement. His fecal alpha 1-antitrypsin level was elevated consistent with protein losing enteropathy. The absence of serological markers of his gut disease was thought to be due to the humoral immune defect even though vaccine responses to *H. influenzae* type B (0.44 μg/ml, protective range >0.15) and tetanus toxoid (0.16 IU/ml protective range >0.1) were preserved. He had low levels of diphtheria antibodies and pneumococcal antibodies, which may have reflected absence of recent immunization. Vaccine challenge responses were not undertaken prior to IVIG treatment, because of the severity of the immune defect. The major immunology results are shown in Table 1.

**Table 1:** Summary of major immunology results

**Serology**	**Patient**	**Reference interval**
IgG	1.5 g/l	>8 g/l
IgA	0.4 g/l	>0.8 g/l
IgM	0.4 g/l	>0.3 g/l
Tetanus antibodies	0.16 IU/ml	>0.1 IU/ml
*H. influenzae* type B antibodies	0.44 μg/ml	>0.15 μg/ml
Diphtheria antibodies	0.01 IU/ml	>0.1 IU/ml
Isohemagglutinins	Present	
Cell markers	Patient	Reference interval
CD4	498 × 10^6^/l	500-1650 × 10^6^/l
CD8	271 × 10^6^/l	210-1200 × 10^6^/l
CD3	794 × 10^6^/l	780-2600 × 10^6^/l
CD19	122 × 10^6^/l	80-600 × 10^6^/l
CD56	24 × 10^6^/l	40-600 × 10^6^/l
Switched memory B cells (CD19+CD27+IgD-)	7.3%	5-21%

**Figure 3:**
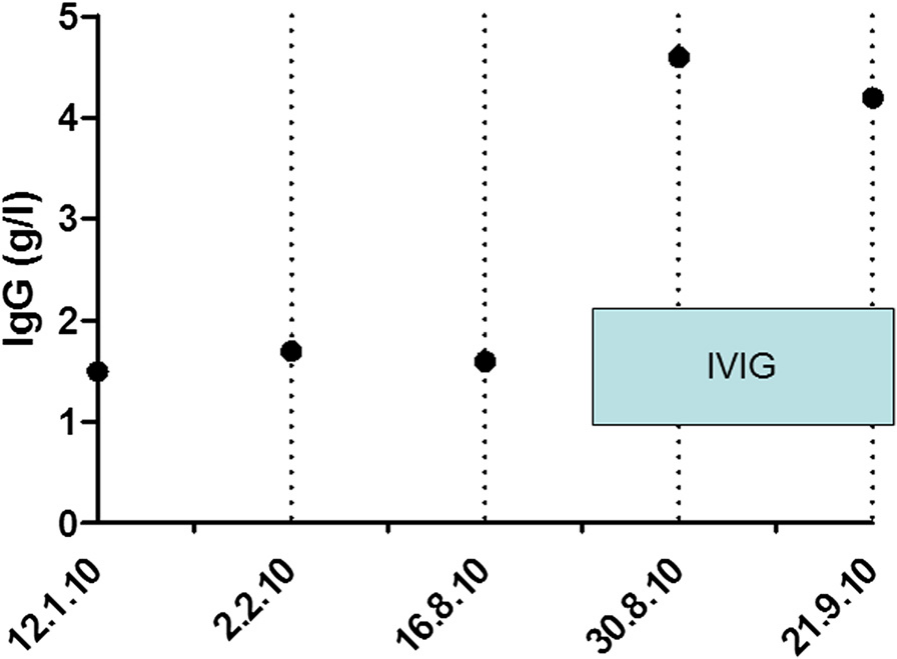
IgG levels before and during IVIG treatment.

Personal history and review of computerized medical records revealed a benign infectious history prior to the current presentation. Repeat testing confirmed persistent severe hypogammaglobulinemia. Immunophenotyping showed normal numbers of B cells (12% of total lymphocytes) and the presence isotype switched memory B cells. He was thought to have protein losing enteropathy secondary to CVID. He was commenced on high dose IVIG but his serum IgG failed to increase to the normal range (Figure 3). In spite of his immunosuppression his albumin remained at 23 g/l. The fecal calprotectin levels remained markedly elevated indicating ongoing bowel inflammation. The clinical immunology service considered the use of Infliximab for the refractory CVID-associated enteritis.

The gastroenterology team became increasingly suspicious about the atypical presentation and lack of response to treatment. A bleeding time was prolonged raising the possibility of NSAID abuse. Careful review of gastric biopsies revealed micro ulcerations. These findings were discussed but both the patient and his wife offered repeated assertions that he had not taken any NSAIDs or similar agents for several months. Subsequent to that appointment however, his family discovered numerous concealed packets of an Ibuprofen/codeine combination and brought this to the attention of the clinical staff.

When confronted with this information, the patient admitted surreptitious use of NSAIDS throughout the preceding year. It was felt by both the gastroenterology and immunology teams that all his symptoms were due to small bowel enteropathy caused by NSAID abuse. His immunosuppression and immunoglobulin infusions were discontinued and per his family, his symptoms abated.

Although the patient declined psychiatric evaluation or outpatient therapy, collateral information elicited a complicated history. His family psychiatric history included bipolar disorder and addiction. In terms of his social history, the patient had undergone more than 6 career changes, including biochemistry and chemistry training as he had indicated at one point that he planned to pursue a career in medicine. According to his family, he frequently read medical texts and at times he described himself as a “pre-med” or a medical student although he had no formal medical training. He also reportedly was prone to self-medication that extended to significant use of recreational drugs such as marijuana, peyote and other hallucinogens that he felt “sharpened his understanding”.

In addition to illegal drugs, family reported that he used caffeine pills and energy drinks on a daily basis. His social history also included ongoing issues with financial stressors. His personal psychiatric history included sexual abuse as a child and a history of at least one known suicide attempt. While he was noted to be impulsive, he was not known to have received a diagnosis or treatment for bipolar disorder. He had been diagnosed and treated for depression. He has not returned for follow up.

## Discussion

This patient illustrates the difficulty in identifying and treating patients with factious disorder. Given the severity of his anemia and bowel ulceration in the setting of the poor response to treatment, a severe gastrointestinal variant of CVID was suspected. His hypogammaglobulinemia proved relatively resistant to treatment with IVIG (Figure 3). He was on the verge of being treated with biological agents including Infliximab when his wife contacted the clinical staff on the discovery of surreptitious NSAID use. Apart from the expense, biological agents expose patients to serious risks including death from infections and malignancy. The self-induced gastrointestinal disease also had the potential for a fatal outcome if he had suffered bowel perforation. Deaths from the self-induced disorder or its treatment have been described in factitious disorder [7,8].

Patients with factitious disorders frequently cause diagnostic confusion and anxiety in clinicians when they fail to improve as expected. They cause anxiety as the treating clinician becomes increasingly concerned about a missed diagnosis and the risk to professional reputation. In some countries the risk of malpractice litigation from a missed diagnosis adds another dimension to the anxiety faced by clinicians, particularly surgeons [9]. This results in a cycle of more invasive and expensive investigations. When the deception is uncovered it can provoke anger and frustration in treating clinicians. Many patients with factitious disorder have traits consistent with Borderline Personality Disorder [10]. With manipulative behaviour, splitting between staff and idealization/denigration, these patients frequently cause significant disharmony and loss of effectiveness in clinical teams [11].

During the course of investigation, three separate diagnoses were entertained in our patient, Celiac disease, Crohn’s disease and CVID. The unifying diagnosis was thought to be CVID, as it is associated with both Crohn’s disease and Celiac disease [12]. In this case, all three possible diagnoses were associated with atypical laboratory results. He did not have tissue transglutaminase, deamidated gliaden peptide antibodies or HLA DQ2 or HLA DQ8 tissue type, making celiac disease unlikely [13]. His histology was not consistent with either Crohn’s disease or celiac disease. When faced with unexpected laboratory results, experienced clinicians are more inclined to believe the patient history in preference to laboratory results. It was the occurrence of multiple inconsistent laboratory results and failure to respond to treatment that eventually raised suspicion of NSAID abuse and factitious disorder, although this was not confirmed until the patient’s family discovered the hidden cache of NSAIDs [14]. It is very likely all three diagnoses were a direct result of NSAID abuse.

As with most patients with factitious disorder, detailed long-term follow up studies were not possible in this case. The albumin increased to 28 g/l two weeks later, after stopping all treatment, indicating improvement of his enteritis.

With the benefit of hindsight CVID was very unlikely in this patient. We have recently proposed new criteria for the diagnosis of CVID (Table 2). In order to meet a diagnosis of probable CVID patients must fulfil the major criteria in category A. They must have symptoms directly attributed to *in vivo* immune system failure (ISF) (category B) and must have supportive laboratory evidence (either category C or D criteria).

**Table 2:** New diagnostic criteria for CVID

A. Must meet all major criteria	
	❖ Hypogammaglobulinemia: IgG below 5 g/L for adults
	❖ No other cause identified for immune defect
	❖ Age > 4 years
B. Clinical sequelae directly attributable to *in vivo* immune system failure (ISF) (1 or more criteria)	
	❖ Recurrent, severe or unusual infections.
	❖ Poor response to antibiotics
	❖ Breakthrough bacterial infections in spite of prophylactic antibiotics
	❖ Infections in spite of immunization with the appropriate vaccine e.g. HPV disease
	❖ Bronchiectasis and/ or chronic sinus disease
	❖ Inflammatory disorders or autoimmunity
C. Supportive laboratory evidence (3 or more criteria)	
	❖ Concomitant deficiency or reduction of IgA (< 0.8 g/l) and/or IgM (< 0.4 g/l)
	❖ Presence of B cells but reduced memory B cell subsets and/or increased CD21 low subsets by flow cytometry
	❖ IgG3 deficiency (< 0.2 g/l)
	❖ Impaired vaccine responses compared to age-matched controls
	❖ Transient responses to vaccines compared to age-matched controls
	❖ Absent isohemagglutinins (if not blood group AB)
	❖ Serological support for autoimmunity in section B e.g. positive Coombs test
	❖ Sequence variations of genes predisposing to CVID e.g. *TACI, BAFFR, MSH5* etc
D. Presence of any one of relatively specific histological markers of CVID (Not required for diagnosis but presence increases diagnostic certainty)	
	❖ Lymphoid interstitial pneumonitis
	❖ Granulomatous disorder
	❖ Nodular regenerative hyperplasia of the liver
	❖ Nodular lymphoid hyperplasia of the gut
	❖ Absence of plasma cells on gut biopsy

Meeting criteria in categories ABC or ABD indicates probable CVID. Patients meeting criteria ABC and ABD should be treated with IVIG/scIG. Patients meeting criteria A alone, AB or AC or AD but not B, are termed possible CVID. Some of these patients may need to be treated with IVIG/scIG. Patients with levels of IgG >5 g/l, not meeting any other criteria are termed hypogammaglobulinemia of uncertain significance (HGUS). A detailed review of these criteria have been recently published [15].

This patient did not meet category C or D criteria. Analysis of his serum immunoglobulin profile of reduced IgG with preserved IgM was compatible with protein-losing enteropathy induced by NSAID abuse. His IgA level returned to the normal range (0.8 g/l, nr > 0.8) just before he was lost to follow up. The majority of CVID patients have a concomitant reduction of IgA and or IgM in addition to reduced IgG levels [15]. Review of his blood transfusion records showed the presence of isohemagglutinins, indicating preserved antibody responses to carbohydrates. The presence of normal numbers of switched memory B cell subsets, although only undertaken on one occasion [16] makes it is unlikely this patient had CVID. We did not measure his IgG3 level and we did not undertake studies of susceptibility genes for CVID [17].

He does not have category D criteria, which are the presence of characteristic histological markers of CVID, such as granulomatous disease [18]. Retrospective review of his intestinal biopsies confirmed the presence of plasma cells, again making CVID very unlikely [15]. He therefore does not meet the proposed new criteria for CVID (Table 2) [15]. Application of these criteria will be helpful in distinguishing CVID from cases of secondary hypogammaglobulinemia.

Patients with factitious disorder are notoriously difficult to treat [19]. When their deception is uncovered, it may provoke anger and patients frequently discharge themselves from medical care. They may continue to seek treatment at other medical institutions (peregrination). There is usually lack of insight and these patients rarely engage in on-going psychotherapy. Long-term follow up studies of large cohorts of such patients are absent from the medical and psychiatric literature [20]. Like many others, our patient had a link to the medical profession [3].

This is the first factitious presentation of a well-defined primary immune deficiency disorder, which we have termed hypogammaglobulinemia factitia. Given the uncanny resemblance to refractory CVID, factitious disorder was not suspected for several months. It is unclear if this patient intended to simulate CVID.

His behaviour does not suggest drug addiction. Although ibuprofen products in New Zealand often contain codeine, it is unlikely that the non-disclosure of the NSAIDs was due to substance abuse as there was no drug seeking behaviour or requests for more potent opiates. If codeine abuse was main issue, there would have been no need for the patient to use a combination drug. Furthermore there was unquestioning willingness – even eagerness - to undergo more invasive and dangerous treatments as has been described in factitious disorder [1].

The family also reported that the patient avidly discussed his ongoing medical care and his evolving medical diagnoses with great interest. He behaved as if he was a member of the medical team because of his previous interest in clinical training. In retrospect, it was noted that he would routinely improve as an in-patient but never when he was at home and that he sought out hospitalization. It is suspected that he assumed the sick role as a maladaptive coping mechanism in the setting of poor emotional coping skills and in the wake of multiple stressors, including an unstable sense of self, family conflict and financial stressors.

In this case, if the patient’s family had not found and reported the NSAID use to the clinical team, it may not have been discovered unless laboratory techniques such as HPLC were deployed to detect NSAID use - despite his denial [21]. Other papers have suggested that the “single stripe sign” on colonscopy may indicate NSAID abuse associated enteropathy, but the sensitivity and specificity of this is not reported and it was not noted in this patient [22].

Patients with factitious disorder can present to any branch of clinical medicine. However if the condition is not considered by general or specialized clinical services, the diagnosis will never be made and expensive and dangerous investigations and treatments will be inappropriately undertaken. Factitious disorder should now be included in the differential diagnosis of severe hypogammaglobulinemia/CVID. Application of the new diagnostic criteria for CVID may allow distinction of CVID from secondary causes of hypogammablobulinemia with greater precision. By reporting this case, we hope that other clinicians will benefit from the inadvertent discovery of this patient’s disorder.

## Consent

The patient described in this report has been lost to follow up. We were not able to obtain written consent for publication of this report. We have presented his case to the Auckland Hospital clinical ethics committee. Approval for publication was given provided all identifying details were removed. Senior legal counsel for Auckland Hospital confirmed no privacy laws were breached by this publication.

## Competing interests

The authors declare they have no competing interests.

## Authors’ contributions

RA wrote the first draft of the publication. PC and SP treated the patient for his gut disorder. They supplied the images. CK substantially revised the manuscript and provided psychiatric data. All authors read and approved the final manuscript.
